# *In Vitro* Antioxidant Properties, HIV-1 Reverse Transcriptase and Acetylcholinesterase Inhibitory Effects of Traditional Herbal Preparations Sold in South Africa

**DOI:** 10.3390/molecules15106888

**Published:** 2010-10-08

**Authors:** Ashwell R. Ndhlala, Jeffrey F. Finnie, Johannes Van Staden

**Affiliations:** Research Centre for Plant Growth and Development, School of Biological and Conservation Sciences, University of KwaZulu-Natal Pietermaritzburg, Private Bag X01, Scottsville 3209, South Africa; E-mails: ndhlala@ukzn.ac.za (A.R.N.); finnie@ukzn.ac.za (J.F.F.)

**Keywords:** acetylcholinesterase, antioxidant, free radicals, herbal products, reverse transcriptase, traditional medicine

## Abstract

The antioxidant potentials for fourteen multipurpose traditional herbal preparations sold in South Africa were determined using the DPPH radical scavenging, ferric reducing power and *β*-carotene-linoleic acid model system, the anti-HIV-1 reverse transcriptase (RT) enzyme inhibitory effects using an ELISA kit and acetylcholinesterase (AChE) enzyme inhibition using the microtitre plate assay. Nine of the herbal mixtures (*Umzimba omubi*, *Umuthi wekukhwehlela ne zilonda*, *Mvusa ukunzi*, *Umpatisa inkosi*, *Imbiza* *ephuzwato*, *Vusa umzimba*, *Supreme one hundred*, *Sejeso herbal mixture Ingwe^®^* and *Ingwe^®^ special muti*) exhibited higher antioxidant potentials, while only four (*Imbiza* *ephuzwato*, *Ingwe^®^ muthi mixture*, *Sejeso herbal mixture Ingwe^®^* and *African potato extract*^TM^) showed potent activity against the RT enzyme. Nine mixtures (*Imbiza* *ephuzwato*, *Umpatisa inkosi*, *African potato extract*^TM^, *Sejeso herbal mixture Ingwe^®^*, *Vusa umzimba*; *Ingwe^®^ muthi mixture*, *Ibhubezi*™, *Lion izifozonke Ingwe^®^* and *Ingwe^®^ special muti*) showed AChE enzyme inhibitory activity greater than 50%. The observed activity exhibited by some of the herbal mixtures gives some credence to the manufacturers’ claims and goes part of the way towards validating their use against certain conditions such as oxidative stress, HIV/AIDS proliferation and some mental conditions. It is however, desirable to carry out further studies to determine the effects of mixing plant species/parts in one mixture on the antioxidant potency as well as isolating active constituents from the herbal mixtures.

## 1. Introduction

Free radicals produced from oxygen to form reactive oxygen species such as the singlet oxygen, superoxide, peroxyl, hydroxyl and peroxynitrite radicals, are constantly produced within living cells for specific metabolic purposes [1]. Living cells have complex mechanisms that act as antioxidant systems to counteract the damaging effects of reactive species. An antioxidant is defined as a substance that, even in small quantities, is able to prevent, or greatly delay, the oxidation of an oxidizable substance [2]. The antioxidant system includes enzymes such as superoxide dismutase, catalase, and glutathione peroxidase. Apart from enzymes, macromolecules such as albumin, ceruloplasmin, and ferritin; and an array of micromolecules, including ascorbic acid, *α*-tocopherol, carotenoids, polyphenols, ubiquinol, reduced glutathione (GSH), methionine, uric acid, and bilirubin also act as protective systems of living cells against oxidation [3]. 

Overproduction of free radicals (oxidative stress), which occurs when the cell’s natural antioxidant systems are overwhelmed, results in severe damage to biological molecules, especially to DNA, proteins and lipids. Oxidative stress has been associated with the progression of chronic conditions such as acquired immune deficiency syndrome (AIDS), cancer, aging, atherosclerosis, inflammation, cardiovascular disease, diabetes, and neurodegenerative diseases such as Parkinson’s and Alzheimer’s diseases [4].

Natural compounds, which are present in herbal products, vegetables, fruits and grains, possess the ability to reduce oxidative damage by acting as antioxidants [5]. The oxidation process proceeds as a chemical reaction that transfers electrons from a reducing substance to an oxidizing agent, forming chain reactions that can be difficult to contain. Antioxidants terminate these chain reactions by removing free radical intermediates, and inhibit other oxidation reactions by getting oxidized [2].

Enzymes are the most attractive targets for drug intervention in the treatment of a number of human diseases. This is because of their involvement in essential catalytic roles in many physiological processes that may be altered during disease progression. The structures of enzymes have lent them well to inhibition by small molecular weight drugs. As a result there is a large and growing interest in the search for compounds that may serve as enzyme inhibitors. Enzyme inhibitors represent almost half the drugs in clinical use at the moment [6].

HIV-1 reverse transcriptase (RT) is a DNA dependent polymerase that catalyses the synthesis of a double stranded DNA copy from a single stranded HIV-RNA. The enzyme is for that reason, essential for the life cycle of HIV-1 and hence a target for anti-HIV therapy [7]. The current therapeutic drugs against HIV infections include the inhibitors of RT and protease enzymes. These include the nucleoside RT inhibitors, nucleotide RT inhibitors and the non-nucleoside RT inhibitors. All non-nucleoside RT inhibitors bind to an allosteric binding pocket (*i.e.* a site other than the enzyme’s active site) [8]. The formation of a RT-non-nucleoside RT inhibitor complex results in short and long range structural changes that make the enzyme inactive [7]. 

Presently, the oral prodrug forms of nucleoside RT inhibitors have been approved for the treatment of HIV infections. A prodrug is a substance that is prescribed and taken orally in an inactive or less active form. Once administered, the prodrug is metabolized *in vivo* into an active form. The use of prodrugs generally increases absorption, distribution and metabolism of most oral pharmaceuticals. The prodrugs include zidovudine, didanosine, lamivudine, stavudine, zalcitabine, abacavir, nevirapine, ritonavir and emtricitabine [8].

Although anti-retroviral drugs have resulted in an improvement of the quality of life amongst HIV infected humans (*i.e.* eating and sleeping well, and keeping a positive outlook on life), the development of resistance, appreciable levels of toxicity, high cost, unavailability and lack of curative effect are their major short-comings [9]. These short-comings, especially the appearance of drug resistant virus strains have resulted in increased efforts for the search of better anti-HIV agents and much attention is now being directed towards natural products [10].

The prevalence of HIV in South Africa is a prominent health concern because the country is believed to have more people infected than in any other country. HIV/AIDS prevalence in South Africa is an epidemic of shattering dimensions with more than five million people being infected by 2002 [9]. The high prevalence of HIV among poor people in South Africa has resulted in an increase in the use of medicinal plants and commercial herbal products. The beneficial effects of herbal preparations and remedies could be due to direct inhibition of HIV replication by blocking RT, boosting of the immune system or curative effects on the opportunistic infections [11].

Acetylcholinesterase (AChE) enzyme that catalyses the hydrolytic degradation of the neurotransmitter acetylcholine, resulting in choline and an acetate group is another pharmacologically important enzyme used in drug development. The enzyme is found at neuromuscular junctions and cholinergic synapses in the central nervous system (CNS), where its activity serves to terminate synaptic transmission [12]. Alzheimer’s disease (AD) that occurs frequently in elderly people is caused by malfunctioning of biochemical pathways associated with the production of the neurotransmitter acetylcholine resulting in progressive memory loss and cognitive impairment [13]. Treatment of AD takes advantage of AChE inhibition. The AChE enzyme has often been implicated in depression and anxiety disorders [14]. Galanthamine, an alkaloid isolated from snowdrop (*Galanthus nivalis*, in the family Amaryllidaceae), is a well known AChE enzyme inhibitor and is widely used for AD treatment [15,16].

Consumption of herbal products, vegetables, fruits and grains has been linked to reduced risk of several conditions, including cancer and cardiovascular diseases. Very little scientific evidence exist that support consumption of herbal preparations in traditional medicine. It is a difficult task to convince the manufacturers or herbalists to reveal the ingredients as well as the recipes of their products as they are well guarded secrets. Fourteen multipurpose commercial herbal mixtures, with various trade names: (1) *Umzimba omubi,* (2) *Umuthi wekukhwehlela ne zilonda,* (3) *Mvusa ukunzi,* (4) *Umpatisa inkosi,* (5) *Imbiza ephuzwato,* (6) *Vusa umzimba,* (7) *Ingwe® muthi mixture,* (8) *Ibhubezi™,* (9) *Supreme one hundred™,* (10) *Sejeso herbal mixture Ingwe®,* (11) *Lion izifozonke Ingwe®,* (12) *Stameta™ BODicare®*, (13) *Ingwe® special muthi* and (14) *African potato extract*^TM^ bought at random from herbal (*muthi*) shops around Pietermaritzburg, KwaZulu-Natal were used in this study. We have previously published the information of these commercial herbal preparations including the manufacturers’ details, claims and directions of use, antibacterial, antifungal, inhibitory effects towards cyclooxygenase enzymes (COX-1 and -2) as well as their mutagenic and cytotoxicty potentials [17,18]. The aims and objectives of this study were to evaluate these fourteen commercial herbal preparations for their antioxidant potentials including the DPPH (2,2–diphenyl–1–picryl hydrazyl) radical scavenging activity, ferric-reducing power and the ability to delay or halt the bleaching of *β*-carotene-linoleic acid in a model system as well as their inhibitory activities against the HIV-1 RT and AChE enzymes.

## 2. Results and Discussion

### 2.1. DPPH radical scavenging assay

The EC_50_ values for the DPPH radical scavenging potentials of the herbal preparations are shown in Table 1. In this study, EC_50_ values less than or equal to 2 mg/mL were considered good activity. The radical scavenging activity of the herbal preparations against DPPH radicals according to the respective EC_50_ values (Table 1) that were less than 2 mg/mL were in the following order: *Ibhubezi*™ > *Lion izifozonke Ingwe^®^* > *Umzimba omubi* > *African potato extract*^TM^ > *Stameta*™ *BODicare^®^* > *Sejeso herbal mixture Ingwe^®^* > *Vusa umzimba* > *Umpatisa inkosi* > *Imbiza ephuzwato*. 

It was interesting to note that some herbal preparations that showed poor activities in our previous study against bacteria and fungi [17], *i.e. Umzimba omubi* and *Umpatisa inkosi*, showed good potential as antioxidants by having higher DPPH radical scavenging percentages as well as lower EC_50_ values. This would validate the use of such herbal preparations as antioxidants in combating diseases. 

A fresh preparation of DPPH radical solution has a deep purple colour which disappears when an antioxidant is added to the medium. Thus, the DPPH radical scavenging assay detects the ability of substances to transfer hydrogen (H) atoms or electron donation via a radical attack (on the DPPH radicals) and convert them to colourless products [19]. Therefore, *Ibhubezi*™, *izifozonke Ingwe^®^*, *Umzimba omubi*, *African potato extract*^TM^, *Stameta*™ *BODicare^®^*, *Sejeso herbal mixture Ingwe^®^*, *Vusa umzimba*, *Umpatisa inkosi* and *Imbiza ephuzwato* may act as antioxidants, probably by donating H-atoms to radicals, thereby terminating free radical chain reactions.

### 2.2. Ferric-reducing power assay

**Figure 1:**
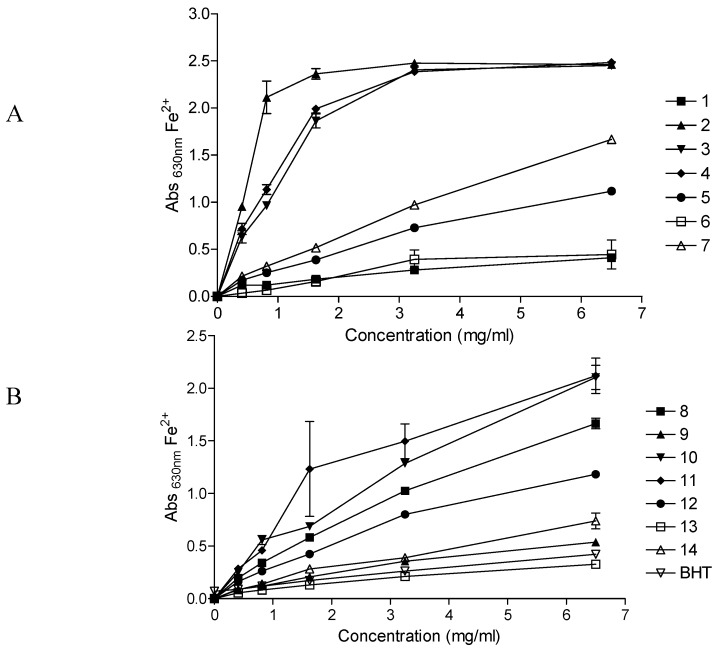
Ferric reducing activity of commercial herbal preparations (A; 1-7 and B; 8-14); BHT- butylated hydroxytoulene. Increase in absorbance of the reaction mixture indicates the increase in reducing power. Refer to Table 1 for the corresponding names of preparations 1-14.

Figure 1 depicts the reducing powers of the fourteen herbal preparations at varying concentrations. The ability of the herbal preparations to reduce the Fe^3+^ solution increased with an increase in concentration of the preparation. Depending on the reducing power of the herbal preparation, the initial yellow colour of the reaction mixture changes to various shades of green and blue. A strong antioxidant (reductant) reduces the Fe^3+^/ferricyanide complex to a green/blue ferrous form, exhibited by higher absorbance values at λ 630 nm. At the highest concentration (6.5 mg/mL) of the herbal preparations, the reducing powers of *Umuthi wekukhwehlela ne zilonda*, *Umpatisa inkosi* and *Mvusa ukunzi* were higher than that of all the other preparations while *Umzimba omubi*, *Vusa umzimba* and *Ingwe^®^ special muti* were weaker than the rest.

As with the DPPH radical scavenging results, herbal preparations which had previously shown poor activities in our previous studies (antimicrobial and inhibition of the cyclooxygenase-1 and -2 enzymes) [17], exhibited higher reduction powers towards the Fe^3+^/ferricyanide complex, *i.e. Umuthi wekukhwehlela ne zilonda*, *Umpatisa inkosi* and *Mvusa ukunzi*. Another interesting observation was that at all concentrations tested, *Imbiza ephuzwato*, which consistently showed higher activities in all the other bioassays in our previous studies (antimicrobial and inhibition of the cyclooxygenase-1 and -2 enzymes) [17], only exhibited moderate reducing power.

### 2.3. β-Carotene-linoleic acid model system (CLAMS)

**Figure 2:**
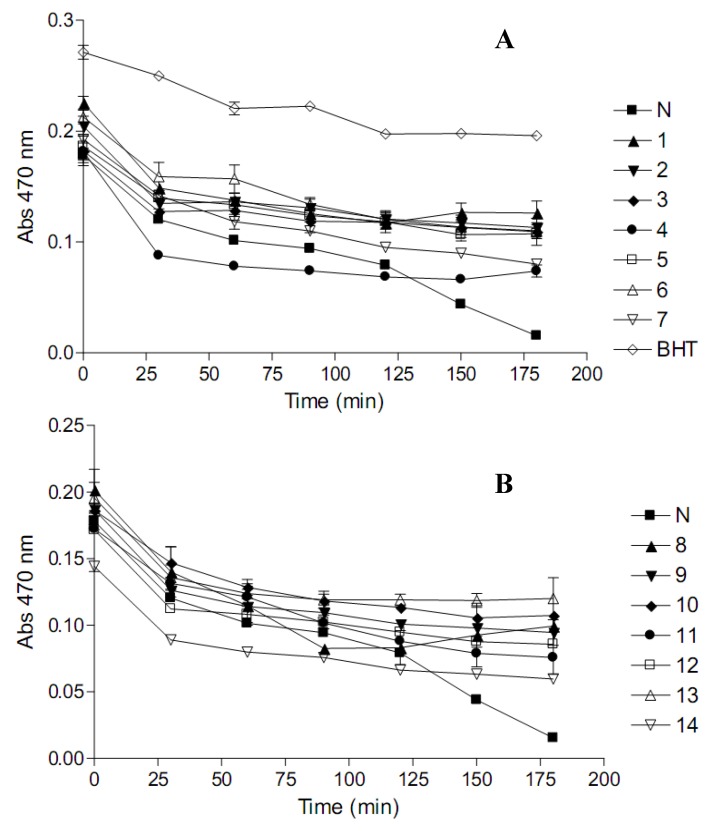
Antioxidant activity of commercial herbal preparations (A; 1-7 and B; 8-14) as determined by the *β*-carotene-linoleic acid coupled oxidation model system. N- negative control (water) and BHT- butylated hydroxytoulene. Slow decrease in absorbance signifies protection of *β*-carotene; hence the test preparation is a potent antioxidant.

The results for the prevention of the heat-induced oxidation of *β*-carotene and linoleic acid in a model system by the herbal preparations are presented in Figure 2. In the presence of an active antioxidant, the rate of *β*-carotene bleaching is reduced. Heat (50 °C)-induced oxidation involves the subtraction of a H-atom from an active methylene group of linoleic acid, forming a linoleate free radical. The linoleate radicals then viciously attack the highly unsaturated *β*-carotene in an effort to regain lost H-atoms. As the *β*-carotene is attacked, it loses its orange colour. The presence of a good antioxidant can prevent the attack on *β*-carotene by neutralizing the linoleate radical.

Nine herbal preparations (*Umzimba omubi*, *Umuthi wekukhwehlela ne zilonda*, *Mvusa ukunzi*, *Imbiza ephuzwato*, *Vusa umzimba*, *Ibhubezi™*, *Sejeso herbal mixture Ingwe^®^* and *Stameta™ BODicare^®^*) exhibited the best potentials to delay the oxidation of β-carotene throughout the incubation period. *African potato extract*^TM^ exhibited the poorest potentials to delay the oxidation of β-carotene. 

Variable antioxidant percentages (ANT), shown in Table 2, were observed for the herbal preparations with *Umzimba omubi*, *Umuthi wekukhwehlela ne zilonda*, *Mvusa ukunzi*, *Umpatisa inkosi*, *Imbiza ephuzwato*, *Vusa umzimba*, *Supreme one hundred*, *Sejeso herbal mixture Ingwe^®^* and *Ingwe^®^* special muti showing moderate activity, between 35% and 70%. *Stameta*™ *BODicare^®^* and *African potato extract*^TM^ exhibited low activity (15% to 35%) while *Ibhubezi™* and *Lion izifozonke* *Ingwe^®^* showed poor activity (<15%).

Lower oxidation rate ratio (ORR) values, like EC_50_ values, denote better antioxidant potentials. Based on ORR, the order of antioxidant capacity with respect to the protection of *β*-carotene against bleaching was as follows; *Mvusa ukunzi* > *Ingwe^®^ special muti* > *Umzimba omubi* > *Umuthi wekukhwehlela ne zilonda* > *Imbiza ephuzwato* > *Sejeso herbal mixture Ingwe^®^* > *Vusa umzimba* > *Supreme one hundred* > *Stameta*™ *BODicare^®^* > *Ingwe^®^* *muthi mixture* > *Lion izifozonke Ingwe^®^* > *Ibhubezi*™ > *African potato extract*^TM^ > *Umpatisa inkosi*.

Many plant secondary metabolites are known to possess antioxidant activity [20]. Flavonoids, one of the largest groups of plant secondary metabolites have been given much credit as being responsible for the antioxidant properties of most plant extracts. Herbal preparations with plant species that are known to contain flavonoids will therefore show high antioxidant potentials. Flavonoids reduce free radicals by quenching, up-regulating or protecting antioxidant defences and chelating radical intermediate compounds. Flavonoids have also been shown to inhibit the enzymes responsible for free radical production, for instance xanthine oxidase, cyclooxygenase, lipoxygenase, microsomal monooxygenase, glutathione *S*-transferase, mitochondrial succinoxidase, NADH oxidase and protein kinase C [21].

However, some compounds could behave as both antioxidants and pro-oxidants, depending on the specific set of conditions such as concentration of the antioxidant and whether oxygen or transition metals are present. In the presence of transition metals, in an aqueous medium, flavonoids auto-oxidize to form highly reactive hydroxyl radicals. Also, some phenolic compounds may act as substrates for xenobiotic metabolizing enzymes such as the cytochrome P450s (CYP450) and peroxidases and other metallo-enzymes, yielding quinone- or quinomethide-type pro-oxidant, mutagenic and alkylating products [18,22].

It is important to use herbal products that exhibit high antioxidant properties such as *Umzimba omubi*, *Umuthi wekukhwehlela ne zilonda*, *Mvusa ukunzi*, *Umpatisa inkosi*, *Imbiza ephuzwato*, *Vusa umzimba*, *Supreme one hundred*, *Sejeso herbal mixture Ingwe^®^* and *Ingwe^®^ special muti* at correct doses and with a controlled diet without a lot of transition metals. There are so many compounds that are responsible for antioxidant properties displayed by the plant extracts, that it becomes important to characterize them.

### 2.4. HIV-1 reverse transcriptase (RT) inhibitory bioassay

**Figure 3:**
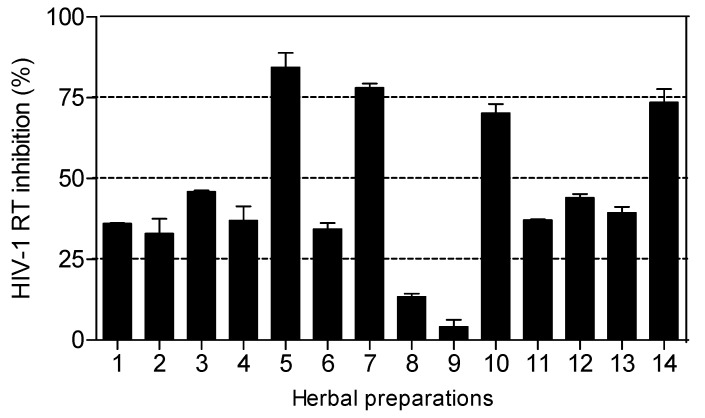
Percentage inhibition of HIV-1 RT by commercial herbal preparations (2.5 mg/mL). Herbal preparations with inhibitory activity above 70% were considered to be highly active. Percentage inhibition by positive controls: Combivir*^®^* (0.5 mg/mL) and Kaletra*^®^* (0.5 mg/mL) were 79.80 ± 0.12 and 62.50 ± 0.31 respectively.

The HIV-1 RT inhibitory activity of the herbal preparations is shown in Figure 3. Only four of the fourteen herbal preparations (*Imbiza ephuzwato*, *Ingwe^®^ muthi mixture*, *Sejeso herbal mixture Ingwe^®^* and *African potato extract*^TM^) showed good activity (>50%) at 2.5 mg/mL. The rest of the preparations showed moderate to low activity.

The IC_50_ values presented in Table 3 show that *Ingwe^®^ muthi mixture* > *Imbiza ephuzwato* > *African potato extract*^TM^ > *Sejeso herbal mixture Ingwe^®^*, in that order, are potent inhibitors of the RT. The mechanisms of action of these four herbal preparations could be through a conformational change on the RT thereby rendering it inactive. It is also possible that the herbal preparations may contain compounds that may act as competitive inhibitors of the RT. Since these preparations are made of a mixture of plant species, there is a possibility that the compounds may be novel. 

In South Africa, there is a rapid proliferation of the consumption of plant based decoctions and herbal preparations by HIV infected people [9], and this is likely to increase as more people become infected and are desperate to get well. The preparation of herbal preparations is cheap and simple; hence they remain a hope for the infected people who cannot access the government sponsored antiretroviral (ARV) programmes.

Based on the results presented in Figure 3 and Table 3, herbal mixtures such as *Imbiza ephuzwato*, *Ingwe^®^ muthi mixture*, *Sejeso herbal mixture Ingwe^®^* and *African potato extract*^TM^ have great potential for use as anti-HIV-1 RT inhibitors. However, more studies are still required to confirm such activity both *in vitro* and *in vivo*. It is therefore important to determine the plant species used to make these four herbal preparations. Upon isolation of active compounds, the mechanisms of action of the herbal mixtures as RT inhibitors will be better understood.

### 2.5. Acetylcholinesterase (AChE) enzyme inhibitory bioassay

**Figure 4:**
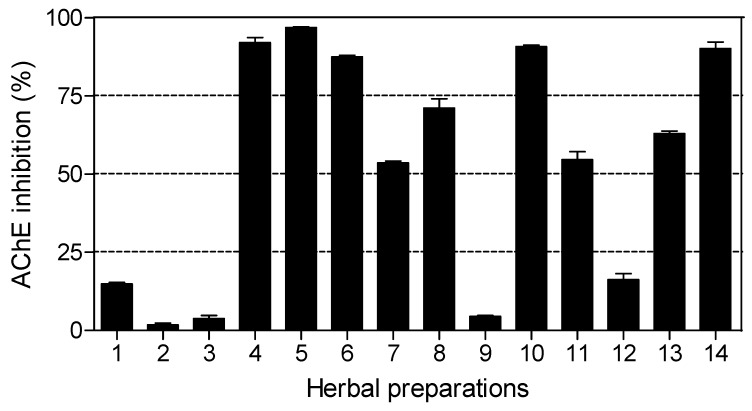
Percentage inhibition of AChE by commercial herbal preparations (1 mg/mL). Herbal preparations with inhibitory activity above 70% were considered to be highly active. Percentage inhibition by galanthamine (20 µM) was 89.90 ± 0.32.

The results of AChE inhibitory activity are presented in Figure 4. Nine out of fourteen herbal preparations showed inhibitory activity above 50%. *Imbiza ephuzwato*, *Umpatisa inkosi*, *African potato extract*^TM^, *Sejeso herbal mixture Ingwe^®^* and *Vusa umzimba* showed good AChE inhibitory activity (>80%). *Ingwe^®^ muthi mixture*, *Ibhubezi*™, *Lion izifozonke Ingwe^®^* and *Ingwe^®^ special muti* exhibited moderate AChE inhibitory activity (between 50% and 70%). The rest of the herbal preparations exhibited very low activity (<20%).

The IC_50_ values for herbal preparations with dose dependent activity are shown in Table 4. Six of the herbal preparations (*Umzimba omubi*, *Umuthi wekukhwehlela ne zilonda*, *Mvusa ukunzi*, *Supreme one hundred* and *Stameta*™ *BODicare^®^*) did not show dose dependant activity, making it not viable to calculate IC_50_ values. The order of potent activity with respect to IC_50_ values was: *African potato extract*^TM^ > *Imbiza ephuzwato* > *Lion izifozonke Ingwe^®^*. 

*Umpatisa inkosi*, a male tonic used as an aphrodisiac and lucky charm showed limited activity against AChE. Herbal preparations used as lucky charms (*Umpatisa inkosi*) are likely to contain plants with psychoactive effects, exerting their therapeutic effects by blocking AChE. This is also possible for the rest of the herbal preparations which showed activity against AChE as they are multipurpose products. Such herbal preparations are likely to contain high levels of alkaloids, known potent inhibitors of AChE. The results of this study indicate that nine out of fourteen herbal preparations offer great potential for the treatment of AD and other psychotic related conditions.

## 3. Experimental

### 3.1. General

Acetylthiocholine iodide (ATCI), 2,2–diphenyl–1–picryl hydrazyl (DPPH), galanthamine, 5,5-dithiobis-2-nitrobenzoic acid (DTNB), AChE enzyme (isolated from electric eels) (type VI-S lyophilized powder) and β–carotene were obtained from Sigma–Aldrich (Sigma Chemical Co., Steinheim, Germany); butylated hydroxytoulene (BHT) and potassium ferricyanide from BDH Chemicals Ltd (Poole, England); trichloroacetic acid, ascorbic acid, polyoxyethylene sorbitan monolaurate (Tween 20), ferric chloride (FeCl_3_) and methanol from Merck KGaA (Darmstadt, Germany); HIV-1 reverse transcriptase colourimetric assay kit was obtained from Roche Diagnostics (Germany). All other chemicals used in the assays were of analytical grade.

Fourteen commercial herbal preparations, *Umzimba omubi*, *Umuthi wekukhwehlela ne zilonda*, *Mvusa ukunzi*, *Umpatisa inkosi*, *Imbiza ephuzwato*, *Vusa umzimba*, *Ingwe^®^* *traditional muthi mixture*, *Ibhubezi*^TM^, *Supreme one hundred*, *Sejeso herbal mixture Ingwe^®^*, *Lion izifozonke Ingwe^®^*, *Stameta*™ *BODicare^®^*, *Ingwe^®^* *special muti* and *African potato extract*^TM^ were bought from herbal shops around Pietermaritzburg, KwaZulu-Natal. 

### 3.2. Sample preparation

The herbal preparations (200 mL) were freeze dried and the resulting material was weighed and resuspended in water and filtered through a sterile 0.22 µm filter unit (Millex® GV, Molsheim, France) to obtain a sterile 50 mg/mL starting concentration and subsequently diluted to lower concentration depending on the assay. *Ingwe^®^ special muti* was obtained from the herbal shop as a powdered material, therefore the extract was prepared following the directions on the packaging. The powdered sample (5 g) was extracted in boiling water (200 mL) with stirring for five minutes (obtaining a tea-like solution) and left to stand until cold. The solution was filtered through Whatman No. 1 and the filtrate was treated as described for the other herbal preparations. 

### 3.3. Bioassays

#### 3.3.1. DPPH radical scavenging activity

The DPPH radical scavenging assay was done as described by Karioti *et al*. [23] with modifications. Fifteen microlitres of each resuspended herbal preparation (0.065, 0.26,0.52, 1.04, 6.25, 12.5, 25 and 50 mg/mL, in triplicate), was diluted in methanol (735 µL) and added to freshly prepared methanolic DPPH solution (750 µL, 50 µM) to give a final volume of 1.5 mL in the reaction mixture. The reaction preparations were prepared under dim light and incubated at room temperature for 30 min in the dark. Absorbance was read at 517 nm using a UV-vis spectrophotometer (Varian Cary 50, Australia), with methanol as the blank solution. A standard antioxidant, ascorbic acid (5, 10, 20, 40, 80 µM) was used as a positive control. A solution with the same chemicals without herbal preparations or standard antioxidants served as the negative control. The assay was repeated twice. The free radical scavenging activity (RSA) as determined by the decolouration of the DPPH solution was calculated according to the formula:

$$\mathrm{RSA}\;\left(\%\right)\;=\;\left\{1\;-\;\left(\frac{{\mathrm{Abs}}_{517\;\mathrm{nm}\;}\;\mathrm{Sample}\;}{{\mathrm{Abs}}_{517\;\mathrm{nm}\;}\;\mathrm{Neg}\;\mathrm{Control}}\right)\right\}\;\times 100$$

where Abs_517_ sample is the absorbance of the reaction mixture containing the resuspended herbal preparation or positive control solution, and Abs_517_ Neg control is the absorbance of the negative control. The EC_50_ (effective concentration) values, representing the amount of extract required to decrease the absorbance of DPPH by 50% was calculated from the percentage radical scavenging activity.

#### 3.3.2. Ferric-reducing power assay

The ferric reducing power of the commercial herbal preparations was determined based on the method by Lim *et al*. [24] with modifications. The ferric-reducing power assay involves the reduction of the Fe^3+^/ferricyanide complex to the ferrous (Fe^2+^) form. Thirty microlitres of each resuspended herbal preparation (6.25 mg/mL) and the positive control (BHT dissolved in methanol) was added to a 96 well microtitre plate in triplicate and two-fold serially diluted down the wells of the plate. To each well, 40 µL potassium phosphate buffer (0.2M, pH 7.2) and 40 µL potassium ferricyanide (1% in phosphate buffer, w/v) were added. The microtitre plate was covered with foil and incubated at 50 °C for 20 min. After the incubation period, 40 µL trichloroacetic acid (10% in phosphate buffer, w/v), 150 µL distilled water and 50 µL FeCl_3_ (0.1% in phosphate buffer, w/v) were added. The microtitre plate was re-covered with foil and incubated at room temperature for 30 min. Absorbance was measured at 630 nm using a microtitre plate reader (Opsys MR^TM^, Dynex Technologies Inc.). The ferric-reducing power of the commercial herbal preparations and ascorbic acid were expressed graphically by plotting absorbance against concentration. The assay was repeated twice.

#### 3.3.3. β-Carotene-linoleic acid model system (CLAMS)

The delay or inhibition of *β*-carotene and linoleic acid oxidation was measured according to the method described by Amarowicz *et al*. [25] with modifications. The antioxidant assay measures the ability of a test solution to prevent or minimize the coupled oxidation of *β*-carotene and linoleic acid in an emulsified aqueous system. In the reaction, the emulsion loses its orange colour due to the reaction with radicals, but this process can be inhibited by antioxidants. 

*β*-Carotene (10 mg) was dissolved in chloroform (10 mL) in a brown Schott bottle. The excess chloroform was evaporated under vacuum, leaving a thin film of *β*-carotene near to dryness. Linoleic acid (200 µL) and Tween 20 (2 mL) were immediately added to the thin film of *β*-carotene and mixed with aerated distilled water (497.8 mL), giving a final *β*-carotene concentration of 20 µg/mL. The mixture was further saturated with oxygen by vigorous agitation to form an orange coloured emulsion. The emulsion (4.8 mL) was dispensed into test tubes to which the resuspended herbal preparation or butylated hydroxytoulene (BHT, 200 µL, 6.25 mg/mL) were added, giving a final concentration of 250 µg/mL in the reaction mixtures. Absorbance for each reaction was immediately (*t = 0*) measured at 470 nm and incubated at 50 °C, with absorbance of each reaction mixture being measured every 30 min for 180 min. Tween 20 solution was used to blank the spectrophotometer. The negative control consisted of 50% methanol in place of the sample. The rate of *β*-carotene bleaching was calculated using the following formula:

$$\mathrm{Rate}\;\mathrm{of}\;\mathrm{bleaching}\;(R)\;=\;\left\{\ln\left(\frac{A_{\;t=0\;}}{A_{\;t=t\;}}\right)\right\}\;\times \frac{1}{t}$$

where *A_t=0_* is the absorbance of the emulsion at 0 min; and *A_t=t_* is the absorbance at time *t* (90 min; any point on the curve can be used for the calculation). The calculated average rates were used to determine the antioxidant activity (ANT) of the respective herbal preparations, and expressed as percent inhibition of the rate of *β*-carotene bleaching using the formula:

$$\%\;\mathrm{ANT}\;=\;\left(\frac{R_{\;\mathrm{control}\;}-{\;R}_{\;\mathrm{sample}\;}\;}{R_{\;\mathrm{control}\;}}\right)\;\times \;100$$

where R_control_ and R_sample_ represent the respective average *β*-carotene bleaching rates for the control and herbal preparation, respectively. Antioxidant activity was further expressed as the oxidation rate ratio (ORR) based on the equation:

$$\mathrm{ORR}\;=\;\frac{R_{\;\mathrm{sample}\;}}{R_{\;\mathrm{control}\;}}$$

#### 3.3.4. HIV-1 reverse transcriptase (RT) inhibitory bioassay

The effect of the herbal preparations on reverse transcription was evaluated using a non-radioactive HIV-RT colourimetric enzyme-linked-immunosorbent serologic assay (ELISA) kit obtained from Roche Diagnostics, Germany. The protocol supplied together with the kit was followed, under nuclease-free conditions. The reverse transcriptase colourimetric assay takes advantage of the ability of RT to synthesize DNA, starting from the template/primer hybrid poly (A) × oligo (dT)15. The kit avoids the use of [3H]- or [32P]-labelled nucleotides which are used for the other classical RT assays. In place of radio-labelled nucleotides, digoxigenin- and biotin-labelled nucleotides are incorporated into one and the same DNA molecule, which is freshly synthesized by the RT. The detection and quantification of synthesized DNA as a parameter for RT activity is followed in a sandwich ELISA protocol: Biotin-labelled DNA freshly synthesized by the RT, binds to the surface of microtitre plate modules (MPM) with wells that were pre-coated with streptavidin. In the next step, an antibody to digoxigenin, conjugated to peroxidase (anti-DIG-POD), binds to the digoxigenin-labelled DNA. In the final step, the peroxidase substrate ABTS (2,2′-azinobis-[3-ethylbenzothiazoline-6-sulfonic acid]-diammonium salt) is added. The peroxidase enzyme catalyzes the cleavage of the substrate, producing a coloured reaction product which is measured spectrophotometrically. 

Three tubes containing water instead of sample were used as negative controls. Another set of three tubes containing lysis buffer and no HIV-1-RT added were included. Combivir® (GlaxoSmithKline) [lamivudine (1.0 mg/mL) + zidovudine (2.0 mg/mL)] and Kaletra® (Abbott) [lopinavir (8.9 mg/mL) + ritonavir (2.2 mg/mL)] were used as positive controls. Results were presented as means ± standard errors of two independent experiments; each experiment was done in duplicate. The IC_50_ (inhibition concentration) values of herbal preparations were calculated using GraphPad Prism (version 4.0) statistical software programme for Windows (GraphPad Software Inc.).

#### 3.3.5. Acetylcholinesterase (AChE) enzyme inhibitory bioassay

Inhibition of AChE enzyme by the herbal preparations was done as described by Ellman *et al.* [26] with some modifications as detailed by Moyo *et al*. [27]. The final concentration of the herbal preparations in the first well containing the highest concentration was 1.0 mg/mL. Galanthamine at 0.12, 0.23, 0.46, 0.92, 1.84, 3.68 and 7.37 µg/mL concentrations and water were used as positive and negative controls respectively. The increase in absorbance due to the spontaneous hydrolysis of the substrate was corrected by subtracting the ratio of reaction before adding the enzyme from the rate after adding the enzyme. Percentage of inhibition was calculated by comparing the reaction rates for the sample to the negative control. Results were presented as means ± standard errors of the experiment in duplicate. The IC_50_ values of herbal preparations were calculated using GraphPad Prism (version 4.0) statistical software programme for Windows (GraphPad Software Inc.).

## 4. Conclusions

The antioxidant potentials, anti-HIV-1 RT and AChE enzyme inhibition were determined for fourteen multipurpose commercial herbal preparations common in South Africa. Nine of the herbal mixtures (*Umzimba omubi*, *Umuthi wekukhwehlela ne zilonda*, *Mvusa ukunzi*, *Umpatisa inkosi*, *Imbiza ephuzwato*, *Vusa umzimba*, *Supreme one hundred*, *Sejeso herbal mixture Ingwe^®^* and *Ingwe^®^ special muti*) exhibited higher antioxidant potentials, while only four (*Imbiza ephuzwato*, *Ingwe^®^ muthi mixture*, *Sejeso herbal mixture Ingwe^®^* and *African potato extract*^TM^) showed potent activity against the RT enzyme. Nine mixtures (*Imbiza ephuzwato*, *Umpatisa inkosi*, *African potato extract*^TM^, *Sejeso herbal mixture Ingwe^®^*, *Vusa umzimba*; *Ingwe^®^ muthi mixture*, *Ibhubezi*™, *Lion izifozonke Ingwe^®^* and *Ingwe^®^ special muti*) showed AChE enzyme inhibitory activity greater than 50%. The observed activity exhibited by some of the herbal mixtures gives some credence to the manufacturers’ claims and goes part of the way to validating their use against certain conditions such as oxidative stress, HIV/AIDS proliferation and some mental conditions. Activity cannot be ruled out in the herbal preparations that showed poor/low activity as they could have different mechanisms of action that were not tested in this study. It is desirable to carry out further studies to determine the effects of mixing plant species/parts in one mixture as well as to try to isolate active constituents. We have already reported on the safety and mutagenic properties of these products.

## Figures and Tables

**Table 1:** DPPH radical scavenging activity (EC50 mg/mL) of fourteen herbal preparations sold in Pietermaritzburg, KwaZulu-Natal.

Herbal preparations	DPPH scavenging activity
	EC_50_ (mg/mL)
*1. Umzimba omubi*	**1.24 ± 0.2**
*2. Umuthi wekukhwehlela ne zilonda*	13.22 ± 0.72
*3. Mvusa ukunzi*	4.52 ± 1.12
*4. Umpatisa inkosi*	**1.86 ± 0.66**
*5. Imbiza ephuzwato*	**1.89 ± 0.90**
*6. Vusa umzimba*	**1.86 ± 0.06**
*7. Ingwe^®^ muthi mixture*	5.48 ± 1.02
*8. Ibhubezi*™	**0.40 ± 0.03**
*9. Supreme one hundred*	21.76 ± 5.46
*10. Sejeso herbal mixture Ingwe^®^*	**1.58 ± 0.08**
*11. Lion izifozonke Ingwe^®^*	**0.94 ± 0.05**
*12. Stameta*™ *BODicare^®^*	**1.41 ± 0.09**
*13. Ingwe^®^ special muti*	4.38 ± 0.02
*14. African potato extract*^TM^	**1.38 ± 0.02**
Ascorbic acid	0.07 ± 0.01

Herbal preparations with EC_50_ values (<2 mg/mL) in bold are considered potent DPPH radical scavengers. The lower the EC_50_, the more rapidly the colour of DPPH radical was being bleached and hence the more potent the antioxidant.

**Table 2:** Antioxidant activity as determined by the *β*-carotene-linoleic acid model system of the fourteen herbal preparations sold in Pietermaritzburg, KwaZulu-Natal.

Herbal preparations	Antioxidant capacity	
	ANT (%)	ORR
*1. Umzimba omubi*	57.14 ± 6.77	0.43 ± 0.07
*2. Umuthi wekukhwehlela ne zilonda*	55.90 ± 4.19	0.44 ± 0.04
*3. Mvusa ukunzi*	60.90 ± 3.62	0.39 ± 0.04
*4. Umpatisa inkosi*	39.16 ± 1.24	1.39 ± 0.10
*5. Imbiza ephuzwato*	51.33 ± 5.79	0.48 ± 0.06
*6. Vusa umzimba*	48.41 ± 5.31	0.52 ± 0.05
*7. Ingwe^®^ muthi mixture*	21.53 ± 8.80	0.78 ± 0.09
*8. Ibhubezi*™	1.69 ± 0.41	1.02 ± 0.39
*9. Supreme one hundred*	39.13 ± 7.40	0.61 ± 0.07
*10. Sejeso herbal mixture Ingwe^®^*	50.31 ± 6.01	0.49 ± 0.06
*11. Lion izifozonke Ingwe^®^*	9.04 ± 2.40	0.91 ± 0.02
*12. Stameta*™ *BODicare^®^*	29.08 ± 6.67	0.71 ± 0.07
*13. Ingwe^®^ special muti*	61.15 ± 4.58	0.39 ± 0.05
*14. African potato extract*^TM^	19.4 ± 2.26	1.19 ± 0.02

ANT (%) - Antioxidant activity calculated on the basis of the rate of *β*-carotene bleaching at *t* = 30, 60 and 90 min. ORR - Oxidation Rate Ratio at *t* = 90. The lower the ORR value, the more protective the herbal preparation against *β*-carotene bleaching.

**Table 3:** HIV-1 RT inhibitory activity (IC_50_ mg/mL) of fourteen herbal preparations sold in Pietermaritzburg, KwaZulu-Natal.

Herbal preparations	HIV-1 RT inhibitory activity
	IC_50_ (mg/mL)
*1. Umzimba omubi*	2.628 ± 0.645
*2. Umuthi wekukhwehlela ne zilonda*	2.762 ± 0.400
*3. Mvusa ukunzi*	3.500 ± 1.605
*4. Umpatisa inkosi*	2.177 ± 0.100
*5. Imbiza ephuzwato*	**0.152 ± 0.001**
*6. Vusa umzimba*	3.493 ± 1.008
*7. Ingwe^®^ muthi mixture*	**0.095 ± 0.008**
*8. Ibhubezi*™	3.983 ± 0.100
*9. Supreme one hundred*	4.320 ± 0.336
*10. Sejeso herbal mixture Ingwe^®^*	**0.367 ± 0.083**
*11. Lion izifozonke Ingwe^®^*	2.389 ± 0.309
*12. Stameta*™ *BODicare^®^*	3.025 ± 0.026
*13. Ingwe^®^ special muti*	2.332 ± 0.764
*14. African potato extract*^TM^	**0.364 ± 0.022**
Combivir*^®^*	0.065 ± 0.003
Kaletra*^®^*	0.330 ± 0.105

Herbal preparations with IC_50_ values in bold are considered potent inhibitors of HIV-1 RT.

**Table 4:** AChE inhibitory activity (IC_50_ mg/mL) of fourteen herbal preparations sold in Pietermaritzburg, KwaZulu-Natal.

Herbal preparations	AChE inhibitory activity
	IC_50_ (mg/mL)
*1. Umzimba omubi*	NC
*2. Umuthi wekukhwehlela ne zilonda*	NC
*3. Mvusa ukunzi*	NC
*4. Umpatisa inkosi*	297.00 ± 5.00
*5. Imbiza ephuzwato*	**0.48 ± 0.01**
*6. Vusa umzimba*	26.50 ± 0.10
*7. Ingwe^®^ muthi mixture*	950 ± 30.00
*8. Ibhubezi*™	367.35 ± 35.85
*9. Supreme one hundred*	NC
*10. Sejeso herbal mixture Ingwe^®^*	21.50 ± 9.50
*11. Lion izifozonke Ingwe^®^*	0.96 ± 0.01
*12. Stameta^™^ BODicare®*	NC
*13. Ingwe^®^ special muti*	NC
*14. African potato extract*^TM^	**0.023 ± 0.01**
Galanthamine	1.6 ± 06 (µM)

Herbal preparations with IC_50_ values in bold are considered potent inhibitors of AChE. NC- IC_50_ could not be calculated because the activity was less than 50% at highest concentration.
